# Enriched Environments in Stroke Units: Defining Characteristics and Limitations

**DOI:** 10.1177/19375867231224972

**Published:** 2024-03-18

**Authors:** Anna Anåker, Maja Kevdzija, Marie Elf

**Affiliations:** 1 School of Health and Welfare, Dalarna University, Falun, Sweden; 2 TU Wien, Department of Building Theory by Design, Faculty of Architecture and Planning, Institute of Architecture and Design, Vienna, Austria

**Keywords:** built environment, enriched environment, rehabilitation, stroke, stroke unit

## Abstract

**Background:**

Individuals with stroke rehabilitated in an enriched environment (EE) compared to a non-EE are more likely to participate in cognitive and social activities, promoting their rehabilitation and well-being. There is a need for a more comprehensive understanding of methods to implement EEs within complex health systems, particularly in stroke units.

**Objective:**

The aim of this systematic review was to compile the concept of an EE in stroke units.

**Methods:**

The literature was sourced from CINAHL, Embase, and Medline databases. A detailed screening and sifting process was used to identify relevant literature. Multiple reviewers independently appraised the identified literature using a Mixed-methods Appraisal Tool. After screening 336 studies, 11 were included.

**Results:**

This review reveals an EE is challenging to define and almost exclusively about activities based on access to individual and communal equipment. Generally, there are no common descriptions or conceptual agreements.

**Conclusions:**

To the best of our knowledge, this is the first study to systematically review the concept of an EE in stroke units and shows that more studies on EEs are needed. The weak definitions and unclear theoretical backgrounds of an EE in the included studies could challenge operationalization. Future research should be based on more precise definitions of an EE and broader interventions that include changes to built and natural environments.

The concept of an enriched environment (EE) has recently gained attention in stroke care. Researchers have sought to translate positive results from animal studies to patients who have suffered from a stroke. In animal models, rats housed in EE show increased neuroplasticity, leading to improved recovery of motor and cognitive function (Deng et al., 2021; Hannan, 2014; Zhan et al., 2020; Zhang et al., 2021). These environments have been enriched with objects stimulating activities, such as running wheels, toys, and social interactions with other rats. In human stroke research, EE has been described as a setting adapted to accommodate the needs of individuals with neurological injury but simultaneously stimulating and challenging to support activities and social contact. EE in humans typically includes books, puzzles, newspapers, games, and music (McDonald et al., 2018). However, factors specific to human environments, such as care organization, culture, and the built environment, may create challenges when translating the concept from animals to humans. Despite the promising results of animal studies, the effectiveness of EEs in improving stroke outcomes in humans remains unclear, and there is no standardized definition of EE (McDonald et al., 2018). Therefore, more rigorous research is needed to evaluate the effectiveness of EEs in human stroke care. Furthermore, there is a need for a more comprehensive understanding of methods to implement an EE within complex health systems, particularly in stroke units. Each system has its own unique organizational structure, culture, and physical environment that may impact the deployment of EE.

***…there is a need for a more comprehensive understanding of methods to implement an EE within complex health systems, particularly in stroke units.***

Stroke is a significant cause of long-term disability (Socialstyrelsen, 2018) and can deteriorate health and increase dependence on daily support, creating challenges for individual patients, caretakers, and society (Langhorne & Ramachandra, 2020). Despite new medical treatment options, many patients suffer from long-term and complex physical and cognitive disabilities (Socialstyrelsen, 2018). Therefore, the need for effective rehabilitation of stroke survivors cannot be underestimated. Recent evidence suggests that early-initiated individually adapted rehabilitation improves mobility, health, and well-being (Langhorne & Ramachandra, 2020). Higher intensity therapy has been found to promote more significant functional improvements during inpatient rehabilitation than less-intensive therapy (Dromerick et al., 2021; Hordacre et al., 2021). Studies have shown that individuals who have suffered from a stroke spend most of their time outside therapy, inactive, alone, and in their bedrooms (Anåker et al., 2017; Shannon et al., 2018). Promoting activity outside of dedicated therapy sessions can be challenging because of the need for assistance with mobilization and exercise, fixed routines in inpatient wards, and the lack of physical therapists outside of therapy hours and on weekends (Clarke et al., 2018). A systematic review reported that stroke survivors spend most of their time outside inactive therapy (median 48.1% of the day), alone (median 53.7% of the day), or in their bedrooms (median 56.5% of the day) (West & Bernhardt, 2012). Barriers to promoting activities outside of dedicated therapy hours have also been reported. After a stroke, a person often requires help in mobilizing and exercising. Furthermore, fixed routines in an inpatient ward, such as rounds, can pose a challenge because of the lack of physical therapists outside therapy hours and on weekends (Bonifacio et al., 2022).

The design of the built environment for stroke units should not be underestimated. The built environment is a place where there are mutual relationships among individuals, care activities, and the surrounding environment, which includes both the built environment (i.e., layout) and plants and nature; furthermore, the design of the built environment affects the patient’s well-being and health (Bernhardt et al., 2022; Ulrich et al., 2018). The design of the built environment can promote health and well-being and is now considered an essential part of high-quality healthcare (Anåker et al., 2016). In line with the Sustainable Development Goals (UN, 2020), the built environment should include universal access to safe, inclusive, accessible, and green public spaces, particularly for women, children, older adults, and individuals with disabilities.

Research on stroke units has shown that built environments can contribute to inactivity and loneliness (Anåker et al., 2019; Hokstad et al., 2015). Opportunities to practice rehabilitation outside therapy sessions and in different inpatient unit locations must be available to intensify rehabilitation further. Individuals with stroke rehabilitated in an EE compared to a non-EE are more likely to participate in cognitive and social activities, promoting their rehabilitation and well-being (McDonald et al., 2018). In research studies, the environment was mainly enriched by creating meeting places (e.g., day rooms and areas in the corridors) with opportunities for activities linked to computers, books, newspapers, or games. Other researchers have made it possible to perform favorite activities like needlework. The results from these studies have shown that EE promotes rehabilitation and well-being (McDonald et al., 2018).

***Individuals with stroke rehabilitated in an EE compared to a non-EE are more likely to participate in cognitive and social activities, promoting their rehabilitation and well-being.***

Together, the above insights suggest that it is crucial to understand and define the enrichment of the environment in the human context, primarily for patients, healthcare professionals, and their work environments. A systematic review would identify not only current definitions and descriptions but could also provide a comprehensive summary of the available evidence regarding the use of EE. This can help identify knowledge gaps, inform future research, and guide clinical practice and policy decisions. This review is an essential step toward advancing the understanding of the potential benefits of EE in stroke units and its implications for stroke care. The compiled knowledge is partly helpful for researchers who plan to implement the concept as well as for decision-makers in planning and designing new stroke units.

## Aim

This review aimed to compile the concept of an EE in stroke units.

## Overarching Research Questions

- What research methods and designs investigate EEs in stroke units?
- How has an EE been described and defined in empirical studies of stroke units?
- What aspects of the EE have been studied?
- What are the interventions and primary outcomes of exposure to an EE for an individual treated in a stroke unit or healthcare staff working in the stroke unit?
- How was the enrichment described concerning the built environment?

## Method

A systematic literature review method was adopted to compile existing empirical studies. This review followed the guidance of the preferred reporting items for systematic reviews and meta-analyses (Page et al., 2021), with the four steps for selecting publications: identification, screening, eligibility, and inclusion.

### Search Strategy and Inclusion/Exclusion Criteria

The research team developed a comprehensive search strategy in consultation with an information specialist with input from the research team, using a combination of free-text terms and key words related to EE and stroke. A literature search was conducted in August 2023 using three electronic databases: CINAHL, Embase, and Medline (Table 1). There were no limitations on the time of publication for the relevant articles since our primary goal was to explore all literature on the EE. The search terms were adjusted according to the rules of the appropriate databases.

**Table 1.** Example of Search String Used for the Systematic Review Process.

Sources: Embase, MEDLINE, Preprints: August 10, 2023
((enrich* NEAR/3 environment*): ti, ab, kw) AND (‘cerebrovascular accident’/exp OR ‘stroke rehabilitation’/de OR ‘cerebrovascular disease’/de OR ‘brain ischemia’/exp OR ‘thromboembolism’/exp OR ‘brain hemorrhage’/exp OR ‘brain infarction’/exp OR ‘brain injury’/de OR stroke$: ti, ab, kw OR poststroke$: ti, ab, kw OR cerebrovasc*: ti, ab, kw OR ‘brain vasc*’: ti, ab, kw OR ‘cerebral vasc*’: ti, ab, kw OR cva*: ti, ab, kw OR sah: ti, ab, kw OR (((brain* OR cerebr* OR cerebell* OR intracran* OR intracerebral) NEAR/2 (isch$emi* OR infarct* OR thrombo* OR emboli* OR occlus*)): ti, ab, kw) OR (((brain* OR cerebr* OR cerebell* OR intracerebral OR intracranial OR subarachnoid) NEAR/2 (h$emorrhage* OR h$ematoma* OR bleed*)): ti, ab, kw)) NOT (‘animal’/exp NOT ‘human’/exp)

The eligibility criteria were based on the population, exposure, and outcome (PEO) framework (Munn et al., 2018; Table 2).

**Table 2.** Population, Exposure, and Outcome (PEO) Framework.

PEO	Description of Inclusion Criteria
Population (P)	Persons (adults and children) treated at a stroke unit, as well as significant others and health professionals at stroke units
Exposure (E)	All empirical studies that discuss, describe, and/or analyze the effects of an enriched environment (exposure) at stroke units on their users (i.e., patients, significant others, and staff). Studies related to nonhuman research/intervention are excluded
Outcomes (O)	All exposures found in studies that discuss, describe, and/or analyze enriched environments on individuals in the stroke units

### Study Selection Process and Data Extraction

**Figure 1.**
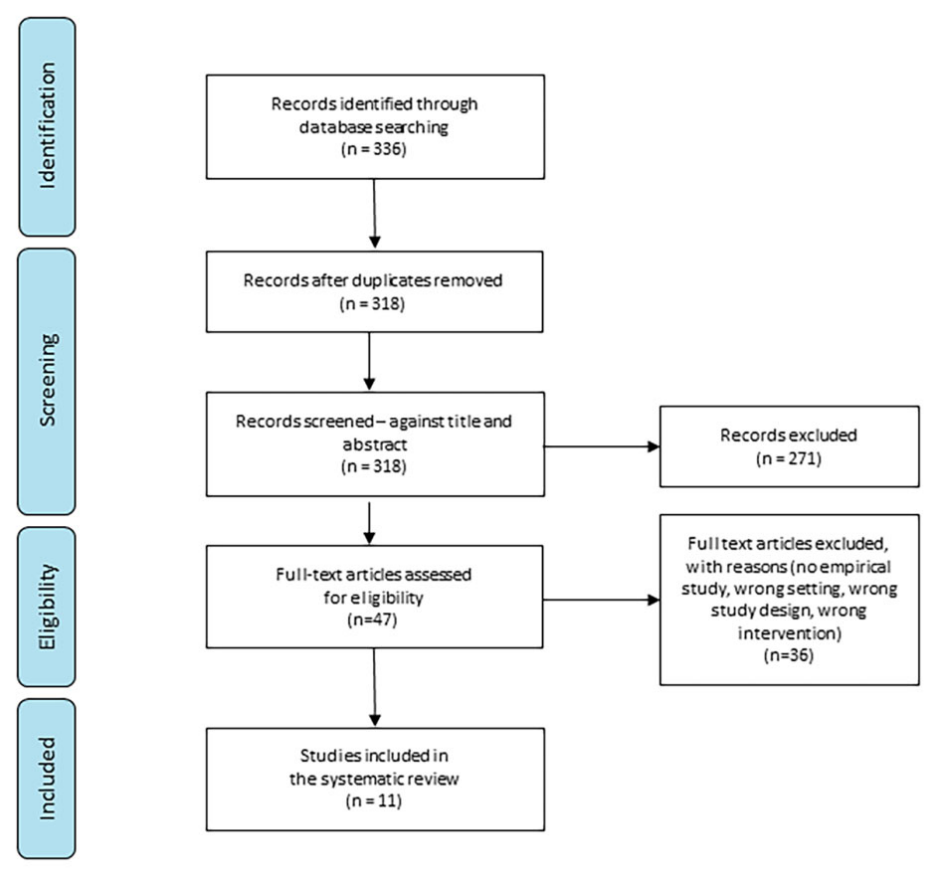
Preferred reporting items for systematic reviews flow diagram of the screening process of the literature (preferred reporting items for systematic reviews and meta-analyses).

All titles were systematically organized using the Covidence (2023) software. Covidence was used to index the research items, exclude duplicated items, screen titles and abstracts, and full-text articles, as well as for data extraction. The first author selected the studies independently. Based on the inclusion criteria described in the PEO framework (Table 2), the selected studies were double-checked by all authors. All authors discussed disagreements at each stage of the selection process until a consensus was reached. During the full-text screening, 36 studies were excluded for the following reasons: not an empirical study, incorrect setting, incorrect study design, and incorrect intervention. After the full-text screening, 11 articles were included for analysis (Figure 1).

A data extraction template was constructed based on the aims and research questions. It included the research method and study design, definition of an EE, type of care, characteristics of the included stroke units (the physical environment), aspects of an EE, primary outcome, and the impact of exposure to an EE. After double-checking by all authors, the extracted data were converted into a Microsoft Excel spreadsheet for analysis.

### Quality Assessment Criteria

The Mixed Methods Appraisal Tool (MMAT; Hong et al., 2018) was used to critically appraise the literature because it facilitates the appraisal of qualitative, quantitative, and mixed methods. The evaluation criteria of the methodological quality of each category of studies differed based on the report of critical methodology issues regarding the specific research types. The MMAT does not provide any guidance on grading the quality of a study. The MMAT tool does not prioritize RCTs/experimental studies over qualitative ones. It is appropriate for systematic reviews to include all studies within an area of knowledge, regardless of the method and study design.

### Data Analysis

The extracted data were analyzed using descriptive statistics in Microsoft Excel 365 (Microsoft Corporation, Redmond, Washington) and qualitatively synthesized via thematic analysis and convergence of terminology (Gough et al., 2012; Thomas & Harden, 2008). In thematic analysis, segments of the text in the articles that addressed the research questions were structured in a matrix and subsequently synthesized in two stages: (1) all included studies were coded line-by-line to form codes from text segments and (2) descriptive themes were developed. Codes were created inductively to capture the meaning of the text segments. The codes were structured as “free codes” without a hierarchical structure. One or more codes were applied to text segments relevant to the research questions. Furthermore, the codes were clustered into related areas to develop descriptive themes (Thomas & Harden, 2008; Table 3).

**Table 3.** Examples of Thematic Analysis: Definition of Enriched Environment (EE).

Author(s)	Quote	Codes	Themes
Janssen et al. (2014)	An EE refers to conditions in which the provision of equipment and organization of the environment facilitates physical, cognitive and social activity (p. 2)	Facilitate	Facilitating patient-driven equipment
Rosbergen et al. (2019)	EE is a multifaceted intervention to create a stimulating housing environment to enhance exploration and engagement in social, cognitive and sensorimotor activities to promote recovery (p. 2)	Stimulating engagement	Increasing engagement in various activities

## Results

After screening 336 studies, 11 were included in the final analysis (Figure 1). All included studies exhibited a quality standard that met the MMAT criteria, with no obvious methodological limitations as appraised using the MMAT. MMAT assessment showed that all included studies had a straightforward clear research question and collected relevant data.

### Characteristics of Studies

We extracted several characteristics of the included studies (Table 4). It appeared that the earliest year an eligible article was published was 2008. This indicates a shift from animal to human studies and an increased interest in this research area in recent years. Nine included studies were from Australia, and two were from Finland. In several cases, these studies were performed by the same research group and/or authors.

### Research Methods and Designs

There was a mix of methods and research designs: of the 11 studies, eight were quantitative and three were qualitative. Various designs were used to collect data on the built environment, including qualitative research (*n* = 3), randomized controlled trials (*n* = 3), and nonrandomized experimental studies (*n* = 5). The participants were primarily patients (nine of the 11 studies). Environmental enrichment was performed in patient bedrooms and communal areas. In two studies, enrichment was applied only to portable equipment, such as an MP3 player, headphones, and listening materials.

**Table 4.** Characteristics of Studies.

First Author/Year of Publication	Country	Title	Aim of the Study	Method	Study Design	Participants	Where Was EE Applied	Description of the Built Environment Before the Intervention
Janssen et al. (2014)	Australia	*An enriched environment increases activity in stroke patients undergoing rehabilitation in a mixed rehabilitation unit: a pilot non-randomized controlled trial*	To determine whether enriching the environment of a mixed rehabilitation unit increased stroke patient activity	Quantitative	Nonrandomized experimental study	Patients	Patient bedroom Communal areas	No
Janssen et al. (2022)	Australia	*Altering the rehabilitation environment to improve stroke survivor activity: A Phase II trial*	To assess the feasibility and safety of a patient-driven model of environmental enrichment for people with stroke undergoing rehabilitation compared with usual rehabilitation	Quantitative	Nonrandomized experimental study	Patients	Patient bedroom Communal areas	No
Khan et al. (2016)	Australia	*An enriched environmental program during inpatient neuro-rehabilitation: A randomized controlled trial*	To assess the effectiveness of an enriched environmental activities program in an inpatient tertiary neuro-rehabilitation unit.	Quantitative	Randomized controlled trial	Patients	Patient bedroom Communal areas	No
Robertson et al. (2020)	Australia	*Acute stroke patients not meeting their nutrition requirements: Investigating nutrition within the enriched environment*	To determine if embedding environmental enrichment that altered the way nutrition was offered and encouraged would result in improved nutrition intake in stroke survivors in an acute stroke unit and to determine if the enriched environment strategies would reduce malnutrition in the acute phase after stroke	Quantitative	Nonrandomized experimental study	Patients	Patient bedroom Communal areas	No
Rosbergen et al. (2017a)	Australia	*Qualitative investigation of the perceptions and experiences of nursing and allied health professionals involved in the implementation of an enriched environment in an Australian acute stroke unit*	To understand perceptions and experiences of nursing and allied health professionals involved in implementing an enriched environment in an acute stroke unit	Qualitative	Qualitative research	Health professionals	Patient bedroom Communal areas	No
Rosbergen et al. (2017b)	Australia	*Embedding an enriched environment in an acute stroke unit increases activity in people with stroke: a controlled before-after pilot study*	To determine if an enriched environment embedded in the acute stroke unit could increase ‘any’ activity, as well as physical, social and cognitive activity levels in stroke patients and to investigate the effect of an enriched environment on functional outcomes, secondary complications and length of stay, and if effect of the intervention was sustained six months after embedding the enriched environment.	Quantitative	Nonrandomized experimental study	Patients	Communal areas	No
Rosbergen et al. (2019)	Australia	*The impact of environmental enrichment in an acute stroke unit on how and when patients undertake activities*	To explore how environmental enrichment had an effect on how and when patient activities were promoted by the enriched compared to control environment and determine the amount of staff assistance provided during patient activities	Quantitative	Nonrandomized experimental study	Patients	Patient bedroom Communal areas	No
Sihvonen et al. (2022)	Finland	*Poststroke enriched auditory environment induces structural connectome plasticity: secondary analysis from a randomized controlled trial*	We hypothesized that vocal music listening would induce longitudinal (baseline to 3-month stage) structural connectivity changes bilaterally in frontotemporal and parietal regions. Furthermore, we hypothesized that also instrumental music would induce similar structural connectivity changes, although less than vocal music in the left-lateralized language-related tracts	Quantitative	Randomized controlled trial	Patients	MP3 player, headphones, and a collection of listening material	No
Särkämö et al. (2008)	Finland	*Music listening enhances cognitive recovery and mood after middle cerebral artery stroke*	To determine whether regular self-directed music listening during the first months after middle cerebral artery (MCA) stroke can enhance the recovery of cognitive functions and mood	Quantitative	Randomized controlled trial	Patients	MP3 player, headphones, and a collection of listening material	No
White et al. (2014)	Australia	*Exploring staff experience of an “enriched environment” within stroke rehabilitation: a qualitative substudy*	To qualitatively explore the experiences of nursing staff involved in a pilot study investigating the feasibility of EE in a rehabilitation ward	Qualitative	Qualitative research	Health professionals	Patient bedroom Communal areas	No
White et al. (2015)	Australia	*Exploring stroke survivor experience of participation in an enriched environment: a qualitative study*	To qualitatively explore stroke survivors experience of implementation of exposure to an EE with typical stroke rehabilitation setting, in order to identify facilitators and barriers to participation	Qualitative	Qualitative research	Patients	Patient bedroom Communal areas	No

### Definition of EE

**Figure 2.**
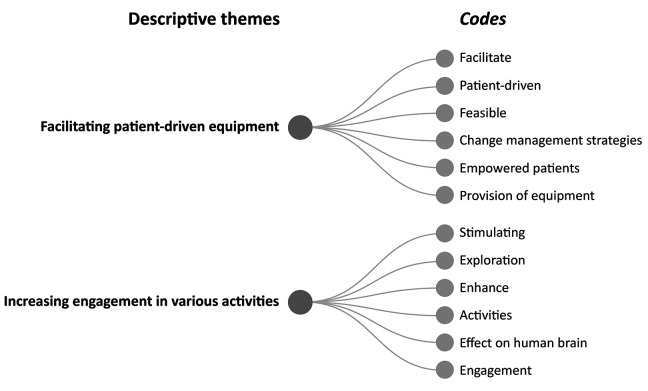
Definition of enriched environment.

All included studies provided a description or definition of the concept of EE. Based on the thematic analysis, enrichment can be divided into two descriptive themes: (1) facilitating patient-driven equipment and (2) increasing engagement in various activities (Figure 2). Studies that defined EE as facilitating patient-driven equipment focused on equipment that would make it easier to facilitate cognitive activities, such as easy access to books, magazines, computers, and games. Furthermore, the equipment helped support patient empowerment during rehabilitation. The studies also argued that adding different types of equipment to the stroke unit could be part of change management strategies:

The model requires minimal resources (i.e., a one-off purchase of equipment and minimal staff involvement) and comprises both communal and individual enrichment and is patient-driven (i.e., patients determine their engagement). (Janssen et al., 2022, p. 2)

The second descriptive theme, increasing engagement in various activities, was based on an enriched, stimulating, exploratory, and engaging environment. For example, an EE was made attractive for patients by providing appealing day rooms with various equipment that drew in the patient and increased physical activity as the patient moved to and from the day room.

### Aspects of the EE

**Figure 3.**
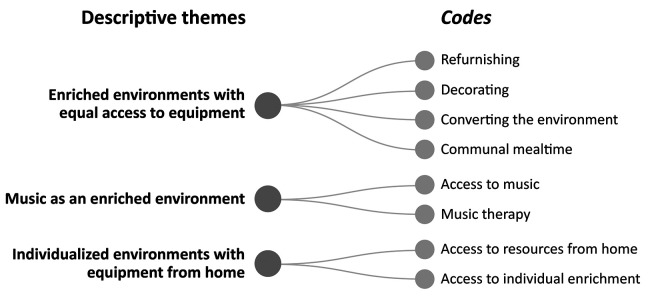
Aspects of the enriched environment.

Various aspects of EEs have been studied, including joint and individual enrichments. These aspects were grouped into three descriptive themes: (1) EEs with equal access to equipment, (2) music as an EE, and (3) individualized environments with equipment from home (Figure 3). The three enrichment groups were primarily based on refurnishing, decorating, and transforming the current environment into a more enriched one. Some studies have established the enrichment of the environment exclusively through various musical interventions:

The music therapist provided the patients with a portable MP3 player, headphones, and a collection of listening material individually selected to match the music or literature preferences of the patient as closely as possible. (Sihvonen et al., 2022, p. 1815)

The third group of studies personalized the environment using home-based equipment:

Family members were encouraged to bring in hobbies and activities that participants enjoyed prior to their stroke. Individual enrichment activities and equipment were stored in a satchel by the participant’s bedside. (Janssen et al., 2014, p. 3)

### Intervention and Primary Outcome of Exposure to the EE

**Figure 4.**
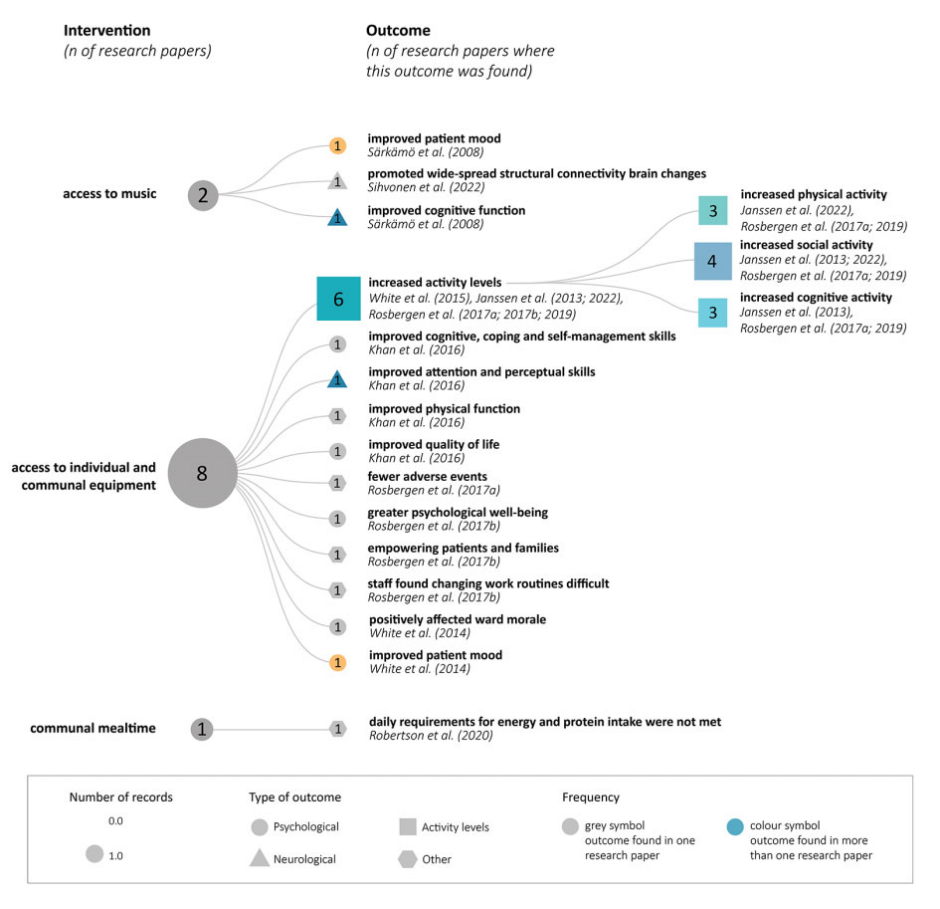
Intervention and primary outcomes.

The focus of the interventions was access to individual and communal enrichment. Two of the included studies only used music interventions for environmental enrichment. These music interventions were based on the patients being given an MP3 player, headphones, or a collection of listening materials. The primary outcome was a wide range of areas. Six studies focused on increased physical, social, and cognitive activity levels. There are several different outcomes for the patients and healthcare staff. Some studies assessed well-being, quality of life, and mood as primary outcomes for patients and staff (Figure 4).

### EE and the Built Environment

None of the included articles described or discussed the built environment before the intervention. Only two included studies mentioned changes related to the built environment as a part of the EE, such as refurnishing and decorating the dining room (White et al., 2014) and transforming public spaces into shared seating for patients and families to encourage social interaction and reduce time in bed (Rosbergen et al., 2019). Even though it is implied that the built environment was modified in these examples, only furniture and its placement changed, not the building itself (layout, size of spaces, spatial connections, access, visibility, materiality, etc.).

## Discussion

This review highlights the challenges in defining EE. While numerous studies have explored the use of equipment to enhance cognitive and physical activities, clear descriptions and definitions of EE are lacking. This lack of a clear and agreed-upon definition and the absence of a theoretical framework make the operationalization of EE challenging. Operationalization of a process reduces subjectivity, minimizes the potential for research bias, and increases study reliability. Therefore, the concept must be clear, explicit, and able to meet the complex needs of users and, above all, patients, healthcare professionals, and significant others.

When adapting the EE concept to animal models, researchers still need to consider the unique factors operating in human environments, such as care organization, culture, and the details of the built environment. Despite the common assumption that EE in healthcare facilities involves modifying the built environment, this is seldom the case for stroke units where EE is implemented. Instead, these interventions are carried out within the physical environment by supplementing them with activities, such as games, computers, and books. Unfortunately, as our review demonstrated, changes in the physical environment are often omitted from descriptions of EE. Only two studies included in our review mentioned modifications related to the built environment, such as refurnishing and decorating the dining room (White et al., 2014) and transforming public spaces into shared seating for patients and families to encourage social interaction and reduce time in bed (Rosbergen et al., 2019). Even in these examples, only furniture and its placement changed, not the environmental layout. All except two studies did not mention any changes to the physical environment of the stroke unit. In the included studies, the environment was primarily enriched through access to activities linked to computers, books, magazines, and games. However, this approach has limitations, and some studies have emphasized the need to redesign the built environment and reorganize therapies and ward areas to provide optimal patient care (Janssen et al., 2022). Additionally, visibility and ease of access to the shared space where enrichment activities are provided from the patient’s room can influence the frequency of visits to this space and, therefore, the use of the provided materials (Anåker et al., 2018; Kevdzija & Marquardt, 2022). This highlights the importance of considering the physical environment when designing and renovating stroke units. Although implementing such interventions may be more expensive than placing some materials in a room, the potential benefits for patients in a stroke unit make it imperative to consider these options.

***When adapting the EE concept to animal models, researchers still need to consider the unique factors operating in human environments, such as care organization, culture, and the details of the built environment.***

Therefore, if a building project aims to develop EE for individuals with stroke, it is critical to address this goal from the initial design process rather than adding it as an afterthought to the finished environment. Moreover, EE can extend from common areas to private rooms because individuals with stroke spend significant time in their rooms. This can be performed at the bedside, as reported by Rosbergen et al. (Rosbergen et al., 2017; Rosbergen et al., 2019) or integrated into the patient room design specifically to promote patients’ physical activity. The successful implementation of this type of intervention will likely depend on the location of the EE and its ability to benefit individuals with stroke, highlighting the need for careful consideration of its placement.

**Figure 5.**
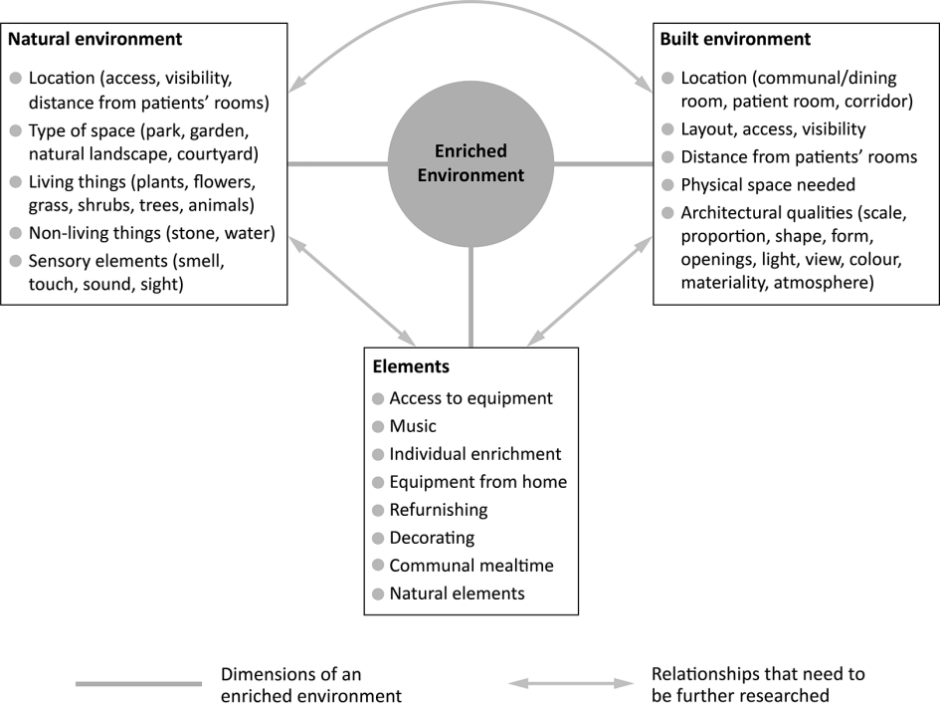
Proposed future research directions.

This review underscores that EE primarily focuses on providing access to individual and communal equipment, with some emphasis on music. However, EE also entails exposure to novel and complex stimuli, which, in the long run, contribute to increased social and physical abilities (Janssen et al., 2018). This perspective is important, and it is crucial to consider whether enrichment can extend beyond equipment-based activities and include cultural and natural environments such as outdoor spaces. The body of scientific evidence showing that natural exposure has beneficial stress/physiological and psychological effects on patients and staff has expanded vastly in recent years (Bratman et al., 2019; Ulrich et al., 2018; Ulrich et al., 2020). Engaging in a natural environment contributes to favorable psychosocial health outcomes. Studies also suggest that the natural environment can contribute to positive emotional and social health outcomes in individuals with stroke (Lakhani et al., 2019). Thus, to fully comprehend the components of EE, additional factors such as the natural environment must be examined. This could promote a deeper understanding of what constitutes an EE in the context of stroke (Figure 5).

***…to fully comprehend the components of EE, additional factors such as the natural environment must be examined.***

Furthermore, various architectural qualities of spaces where environmental enrichment is offered, such as scale and proportion, shape and form, openings, light, view, color, materiality, and atmosphere, might affect how the provided materials are used. To date, no studies have investigated the impact of specifically built environment aspects on the use and efficacy of EE. From the beginning, it is also uncertain how EE may be integrated into the design of a built environment. Based on recent studies demonstrating the importance of the built environment in stroke rehabilitation (Anåker et al., 2019; Anåker et al., 2018; Bernhardt et al., 2004; Hokstad et al., 2015), further research should aim to include different built environment features to investigate the impact of environmental enrichment.

We argue that the EE is an essential aspect of the design of the built environment in the stroke unit. However, several factors need to be considered more closely before the usefulness of an EE can be adequately evaluated, including (i) a comprehensive theoretical framework capable of defining what an EE includes in the context of stroke units and (ii) operationally defining and standardizing EEs across studies within the area of stroke units.

### Strengths and Limitations

This review is the first to comprehensively compile EE research in stroke units. The inclusion of both qualitative and quantitative methodologies strengthens the validity of the research in the field (Polit & Beck, 2021). The authors had ongoing discussions to minimize the risk of bias in the analysis. However, this study has some limitations. First, it was challenging to compare outcomes and effects. Even still, the primary objective of this review was not to compare effects but to compile research on EE. Therefore, the studies included in this review showed modest effects (Janssen et al., 2022) or no improvement (Robertson et al., 2020). Second, comparing studies that use different methods, such as quantitative and qualitative, may be challenging. However, using the MMAT, we could critically appraise the literature as it facilitates the appraisal of qualitative, quantitative, and mixed-method studies without prioritizing RCTs/experimental studies (Hong et al., 2018).

## Conclusion

After analyzing the available research on EE in stroke units, this review highlights the need for more rigorous research in this area. While the current study provides evidence of the benefits of EE, it primarily focuses on easy-to-understand interventions related to individual and communal equipment. To achieve a more precise and agreed-upon definition of EE that encompasses a broader range of interventions, such as changes to built and natural environments, it is crucial to establish a consensus between patients, healthcare professionals, and professionals involved in the planning process who can collectively define and describe what EE means for a stroke unit. In conclusion, this review underscores the need for more extensive and multidisciplinary research to establish the efficacy of EEs in stroke units and their potential benefits to individuals with stroke.

## Implications for Practice

- To be useful in the context of stroke units, the concept of an EE should be underpinned by a comprehensive theoretical framework capable of defining its components and parameters.
- An EE in stroke units includes a broader range of interventions, such as changes to built and natural environments.
- This study highlights the need for a more extensive and multidisciplinary research effort to establish the efficacy of EEs in stroke units and the potential benefits they offer to individuals with stroke.

## References

[bibr1-19375867231224972] Anåker A. Heylighen A. Nordin S. Elf M. (2016). Design quality in the context of healthcare environments: A scoping review. Health Environments Research & Design Journal, 10(4), 136–150. 28643560 10.1177/1937586716679404 PMC5484461

[bibr2-19375867231224972] Anåker A. von Koch L. Heylighen A. Elf M. (2019). “It’s lonely”: Patients’ experiences of the physical environment at a newly built stroke unit. Health Environments Research & Design Journal, 12(3), 141–152. 10.1177/1937586718806696 30336696 PMC6637812

[bibr3-19375867231224972] Anåker A. von Koch L. Sjostrand C. Bernhardt J. Elf M. (2017). A comparative study of patients’ activities and interactions in a stroke unit before and after reconstruction-the significance of the built environment. PLoS One, 12(7), e0177477. 10.1371/journal.pone.0177477 28727727 PMC5519004

[bibr4-19375867231224972] Anåker A. von Koch L. Sjostrand C. Heylighen A. Elf M. (2018). The physical environment and patients’ activities and care: A comparative case study at three newly built stroke units. Journal of Advanced Nursing, 74(8), 1919–1931. 10.1111/jan.13690 29676493

[bibr5-19375867231224972] Bernhardt J. Dewey H. Thrift A. Donnan G. (2004). Inactive and alone: Physical activity within the first 14 days of acute stroke unit care. Stroke, 35(4), 1005–1009. 10.1161/01.str.0000120727.40792.40 14988574

[bibr6-19375867231224972] Bernhardt J. Lipson-Smith R. Davis A. White M. Zeeman H. Pitt N. Shannon M. Crotty M. Churilov L. Elf M. ; on behalf of the NOVELL Redesign collaboration. (2022). Why hospital design matters: A narrative review of built environments research relevant to stroke care. International Journal of Stroke, 17(4), 370–377. 10.1177/17474930211042485 34427477 PMC8969212

[bibr7-19375867231224972] Bonifacio G. B. Ward N. S. Emsley H. C. A. Cooper J. Bernhardt J. (2022). Optimising rehabilitation and recovery after a stroke. Practical Neurology, 22(6), 478–485. 10.1136/practneurol-2021-003004 35896376

[bibr8-19375867231224972] Bratman G. N. Anderson C. B. Berman M. G. Cochran B. de Vries S. Flanders J. Folke C. Frumkin H. Gross J. J. Hartig T. Kahn P. H. Jr Kuo M. Lawler J. J. Levin P. S. Lindahl T. Meyer-Lindenberg A. Mitchell R. Ouyang Z. Roe J. … Daily G. C . (2019). Nature and mental health: An ecosystem service perspective. Science Advances, 5(7), eaax0903. 10.1126/sciadv.aax0903 PMC6656547 31355340

[bibr9-19375867231224972] Clarke D. J. Burton L. J. Tyson S. F. Rodgers H. Drummond A. Palmer R. Hoffman A. Prescott M. Tyrrell P. Brkic L. Grenfell K. Forster A. (2018). Why do stroke survivors not receive recommended amounts of active therapy? Findings from the ReAcT study, a mixed-methods case-study evaluation in eight stroke units. Clinical Rehabilitation, 32(8), 1119–1132. 10.1177/0269215518765329 29582712 PMC6068965

[bibr10-19375867231224972] Covidence. (2023). Covidence—Better systematic review management. covidence.org

[bibr11-19375867231224972] Deng Y-H. Dong L-L. Zhang Y-J. Zhao X-M. He H-Y. (2021). Enriched environment boosts the post-stroke recovery of neurological function by promoting autophagy. Neural Regeneration Research, 16(5), 813–819. 10.4103/1673-5374.297084 33229714 PMC8178758

[bibr12-19375867231224972] Dromerick A. W. Geed S. Barth J. Brady K. Giannetti M. L. Mitchell A. Edwardson M. A. Tan M. T. Zhou Y. Newport E. L. Edwards D. F. (2021). Critical Period After Stroke Study (CPASS): A phase II clinical trial testing an optimal time for motor recovery after stroke in humans. Proceedings of the National Academy of Sciences, 118(39), e2026676118. 10.1073/pnas.2026676118 PMC8488696 34544853

[bibr13-19375867231224972] Gough D. Oliver S. Thomas J. (Ed.). (2012). An introduction to systematic reviews. Sage. https://go.exlibris.link/VZzv03jN

[bibr14-19375867231224972] Hannan A. J. (2014). Environmental enrichment and brain repair: Harnessing the therapeutic effects of cognitive stimulation and physical activity to enhance experience-dependent plasticity. Neuropathol Appl Neurobiol, 40(1), 13–25. 10.1111/nan.12102 24354721

[bibr15-19375867231224972] Hokstad A. Indredavik B. Bernhardt J. Ihle-Hansen H. Salvesen O. Seljeseth Y. M. Schuler S. Engstad T. Askim T. (2015). Hospital differences in motor activity early after stroke: A comparison of 11 Norwegian stroke units. Journal of Stroke and Cerebrovascular Diseases, 24(6), 1333–1340. 10.1016/j.jstrokecerebrovasdis.2015.02.009 25906937

[bibr16-19375867231224972] Hong Q. N. Gonzalez-Reyes A. Pluye P. (2018). Improving the usefulness of a tool for appraising the quality of qualitative, quantitative and mixed methods studies, the Mixed Methods Appraisal Tool (MMAT). Journal of Evaluation in Clinical Practice, 24(3), 459–467. 10.1111/jep.12884 29464873

[bibr17-19375867231224972] Hordacre B. Austin D. Brown K. E. Graetz L. Pareés I. De Trane S. Vallence A. M. Koblar S. Kleinig T. McDonnell M. N. Greenwood R. Ridding M. C. Rothwell J. C. (2021). Evidence for a window of enhanced plasticity in the human motor cortex following ischemic stroke. Neurorehabilitation and Neural Repair, 35(4), 307–320. 10.1177/1545968321992330 33576318 PMC7610679

[bibr18-19375867231224972] Janssen H. Bernhardt J. Walker F. R. Spratt N. J. Pollack M. Hannan A. J. Nilsson M . (2018). Environmental enrichment: Neurophysiological responses and consequences for health. In Bird W. Bosch M. van den (Eds.), Oxford textbook of nature and public health: The role of nature in improving the health of a population (pp. 71–78). Oxford University Press.

[bibr19-19375867231224972] Janssen H. Ada L. Bernhardt J. McElduff P. Pollack M. Nilsson M. Spratt N. J. (2014). An enriched environment increases activity in stroke patients undergoing rehabilitation in a mixed rehabilitation unit: A pilot non-randomized controlled trial. Disability and Rehabilitation, 36(3), 255–262. 10.3109/09638288.2013.788218 23627534

[bibr20-19375867231224972] Janssen H. Ada L. Middleton S. Pollack M. Nilsson M. Churilov L. Blennerhassett J. Faux S. New P. McCluskey A. Spratt N. J. Bernhardt J. ; On Behalf of the AREISSA Trial Group. (2022). Altering the rehabilitation environment to improve stroke survivor activity: A Phase II trial. International Journal of Stroke, 17(3), 299–307. 10.1177/17474930211006999 33739202

[bibr22-19375867231224972] Kevdzija M. Marquardt G. (2022). Stroke patients’ nonscheduled activity during inpatient rehabilitation and its relationship with the architectural layout: A multicenter shadowing study. Topics in Stroke Rehabilitation, 29(1), 9–15. 10.1080/10749357.2020.1871281 33423616

[bibr222-19375867231224972] Khan F. Amatya B. Elmalik A. Lowe M. Ng L. Reid I. Galea M. P. (2016). An enriched environmental programme during inpatient neuro-rehabilitation: A randomized controlled trial. J Rehabil Med, 48(5), 417–425. 10.2340/16501977-2081 27058405

[bibr23-19375867231224972] Lakhani A. Norwood M. Watling D. P. Zeeman H. Kendall E. (2019). Using the natural environment to address the psychosocial impact of neurological disability: A systematic review. Health & Place, 55, 188–201. 10.1016/j.healthplace.2018.12.002 30583914

[bibr24-19375867231224972] Langhorne P. Ramachandra S. ; Stroke Unit Trialists’ Collaboration. (2020). Organised inpatient (stroke unit) care for stroke: Network meta-analysis. Cochrane Database of Systematic Reviews, 4(4), Cd000197. 10.1002/14651858.CD000197.pub4 32324916 PMC7197653

[bibr25-19375867231224972] McDonald M. W. Hayward K. S. Rosbergen I. C. M. Jeffers M. S. Corbett D. (2018). Is environmental enrichment ready for clinical application in human post-stroke rehabilitation? Frontiers in Behavioral Neuroscience, 12, 135. 10.3389/fnbeh.2018.00135 30050416 PMC6050361

[bibr26-19375867231224972] Munn Z. Stern C. Aromataris E. Lockwood C. Jordan Z. (2018). What kind of systematic review should I conduct? A proposed typology and guidance for systematic reviewers in the medical and health sciences. BMC Medical Research Methodology, 18(1), 5. 10.1186/s12874-017-0468-4 29316881 PMC5761190

[bibr27-19375867231224972] Page M. J. McKenzie J. E. Bossuyt P. M. Boutron I. Hoffmann T. C. Mulrow C. D. Shamseer L. Tetzlaff J. M. Akl E. A. Brennan S. E. Chou R. Glanville J. Grimshaw J. M. Hróbjartsson A. Lalu M. Li T. Loder E. W. Mayo-Wilson E. McDonald S. … Moher D . (2021). The PRISMA 2020 statement: An updated guideline for reporting systematic reviews. BMJ, 372, n71. 10.1136/bmj.n71 PMC8005924 33782057

[bibr28-19375867231224972] Polit D. F. Beck C. T . (2021). Nursing Research: Generating and assessing evidence for nursing practice. Wolters Kluwer.

[bibr29-19375867231224972] Robertson S. T. Grimley R. S. Anstey C. Rosbergen I. C. (2020). Acute stroke patients not meeting their nutrition requirements: Investigating nutrition within the enriched environment. Clinical Nutrition, 39(5), 1470–1477. 10.1016/j.clnu.2019.06.009 31235416

[bibr30-19375867231224972] Rosbergen I. C. M. Brauer S. G. Fitzhenry S. Grimley R. S. Hayward K. S. (2017a). Qualitative investigation of the perceptions and experiences of nursing and allied health professionals involved in the implementation of an enriched environment in an Australian acute stroke unit. BMJ Open, 7(12), e018226. 10.1136/bmjopen-2017-018226 PMC5778299 29273658

[bibr300-19375867231224972] Rosbergen I. C. Grimley R. S. Hayward K. S. Walker K. C. Rowley D. Campbell A. M. McGufficke S. Robertson S. T. Trinder J. Janssen H. Brauer S. G. (2017b). Embedding an enriched environment in an acute stroke unit increases activity in people with stroke: a controlled before-after pilot study. Clin Rehabil, 31(11), 1516–1526. 10.1177/0269215517705181 28459184

[bibr31-19375867231224972] Rosbergen I. C. M. Grimley R. S. Hayward K. S. Brauer S. G. (2019). The impact of environmental enrichment in an acute stroke unit on how and when patients undertake activities. Clinical Nutrition, 33(4), 784–795. 10.1177/0269215518820087 30582368

[bibr32-19375867231224972] Shannon M. M. Elf M. Churilov L. Olver J. Pert A. Bernhardt J. (2018). Can the physical environment itself influence neurological patient activity? Disability and Rehabilitation, 41(10), 1117–1189. 10.1080/09638288.2017.1423520 29343110

[bibr33-19375867231224972] Sihvonen A. J. Soinila S. Sarkamo T. (2022). Post-stroke enriched auditory environment induces structural connectome plasticity: Secondary analysis from a randomized controlled trial. Brain Imaging and Behavior, 16(4), 1813–1822. 10.1007/s11682-022-00661-6 35352235 PMC9279272

[bibr34-19375867231224972] Socialstyrelsen. (2018). Nationella riktlinjer för vård vid stroke. Swedish national guidelines for stroke care. Socialstyrelsen.

[bibr401-19375867231224972] Särkämö T. Tervaniemi M. Laitinen S. Forsblom A. Soinila S. Mikkonen M. Autti T. Silvennoinen H. M. Erkkilae J. Laine M. Peretz I. Hietanen M. (2008). Music listening enhances cognitive recovery and mood after middle cerebral artery stroke. Brain, 131, 866–876. 10.1093/brain/awn013 18287122

[bibr35-19375867231224972] Thomas J. Harden A. (2008). Methods for the thematic synthesis of qualitative research in systematic reviews. BMC Medical Research Methodology, 8, 45. 10.1186/1471-2288-8-45 18616818 PMC2478656

[bibr36-19375867231224972] Ulrich R. S. Bogren L. Gardiner S. K. Lundin S. (2018). Psychiatric ward design can reduce aggressive behavior. Journal of Environmental Psychology, 57, 53–66. 10.1016/j.jenvp.2018.05.002

[bibr37-19375867231224972] Ulrich R. S. Cordoza M. Gardiner S. K. Manulik B. J. Fitzpatrick P. S. Hazen T. M. Perkins R. S. (2020). ICU patient family stress recovery during breaks in a hospital garden and indoor environments. Health Environments Research & Design Journal, 13(2), 83–102. 10.1177/1937586719867157 31390887

[bibr38-19375867231224972] UN. (2020). Transforming our world: The 2030 agenda for sustainable development. https://sustainabledevelopment.un.org/content/documents/21252030%20Agenda%20for%20Sustainable%20Development%20web.pdf

[bibr39-19375867231224972] West T. Bernhardt J. (2012). Physical activity in hospitalised stroke patients. Stroke Research and Treatment, 2012, 813765. 10.1155/2012/813765 21966599 PMC3182066

[bibr40-19375867231224972] White J. H. Alborough K. Janssen H. Spratt N. Jordan L. Pollack M. (2014). Exploring staff experience of an “enriched environment” within stroke rehabilitation: A qualitative sub-study. Disability and Rehabilitation, 36(21), 1783–1789. 10.3109/09638288.2013.872200 24369101

[bibr400-19375867231224972] White J. H. Bartley E. Janssen H. Jordan L. A. Spratt N. (2015). Exploring stroke survivor experience of participation in an enriched environment: a qualitative study. Disabil Rehabil, 37(7), 593–600. 10.3109/09638288.2014.935876 25754445

[bibr41-19375867231224972] Zhan Y. Li M. Z. Yang L. Feng X. F. Lei J. F. Zhang N. Zhao Y. Y. Zhao H. (2020). The three-phase enriched environment paradigm promotes neurovascular restorative and prevents learning impairment after ischemic stroke in rats. Neurobiology of Disease, 146, 105091. 10.1016/j.nbd.2020.105091 32979506

[bibr42-19375867231224972] Zhang X. Yuan M. Yang S. B. Chen X. Y. Wu J. C. Wen M. Y. Yan K. Bi X. (2021). Enriched environment improves post-stroke cognitive impairment and inhibits neuroinflammation and oxidative stress by activating Nrf2-ARE pathway. International Journal of Neuroscience, 131(7), 641–649. 10.1080/00207454.2020.1797722 32677581

